# Pain Management in People with Diabetes-Related Chronic Limb-Threatening Ischemia

**DOI:** 10.1155/2021/6699292

**Published:** 2021-05-08

**Authors:** Xiaoyan Jiang, Yi Yuan, Yu Ma, Miao Zhong, Chenzhen Du, Johnson Boey, David G. Armstrong, Wuquan Deng, Xiaodong Duan

**Affiliations:** ^1^Department of Endocrinology, Diabetic Foot Center, Chongqing University Central Hospital, Chongqing Emergency Medical Center, Chongqing 400014, China; ^2^Department of Podiatry, National University of Hospital Singapore, Singapore 169608; ^3^Keck School of Medicine of University of Southern California, Los Angeles, CA 90033, USA; ^4^Department of Rehabilitation, The Affiliated Hospital of Southwest Medical University, Luzhou, Sichuan 646000, China

## Abstract

Management of neuropathic pain in people with diabetes has been widely investigated. However, little attention was paid to address ischemic-related pain in patients with diabetes mellitus who suffered from chronic limb-threatening ischemia (CLTI), the end stage of lower extremity arterial disease (LEAD). Pain management has a tremendous influence on patients' quality of life and prognosis. Poor management of this type of pain owing to the lack of full understanding undermines patients' physical and mental quality of life, which often results in a grim prognosis, such as depression, myocardial infarction, lower limb amputation, and even mortality. In the present article, we review the current strategy in the pain management of diabetes-related CLTI. The endovascular therapy, pharmacological therapies, and other optional methods could be selected following comprehensive assessments to mitigate ischemic-related pain, in line with our current clinical practice. It is very important for clinicians and patients to strengthen the understanding and build intervention strategy in ischemic pain management and possible adverse consequence.

## 1. Introduction

Lower extremity arterial disease (LEAD) in diabetes is a leading cause of limb loss and has a profoundly negative impact on quality of life and early mortality [1]. Although intermittent claudication (IC) is considered to be the early symptom in patients with LEAD, it could be relieved by exercise, pharmacotherapy, and quitting smoking [2]. By contrast, critical limb ischemia (CLI) represents the end-stage manifestation of LEAD, with a major amputation rate of 30%, mortality rate of 25%, and chronic pain of 20% at one year [3, 4].

Although the pain is an important issue for most patients with CLI, it is often poorly managed and mismanaged [5]. Many individuals with LEAD not only have a higher amputation rate and mortality but also experience ischemic pain [6–8]. It has been widely established that coronary artery disease (CAD) and diabetes mellitus (DM) are among the prevailing comorbidities in patients with peripheral arterial disease (PAD). However, CLTI is observed to have higher mortality rate than symptomatic CAD [9].

Moreover, ischemic ulcers carried higher mortality risk than neuropathic ulcers in patients with DM, although neuropathic ulcers induce considerable morbidity than ischemic ulcers [10]. In addition, LEAD independently increases the risk of diabetes-related anxiety and depression with a negative attitude to treatment, which often leads to poor healing and amputation [11, 12].

Presently, no randomized clinical trial has been conducted, and no specific practice recommendation has been provided in the management of ischemic pain in patients with CLI [5]. It is difficult to conduct a systematic review and meta-analysis because widespread reviews of the literature or randomized controlled trials focused on pain management in CLI are scarce, especially in people with diabetes. The timing and means of the good treatment protocol for CLI patients are very important to the patients because they often determine the success rate of limb salvage.

Therefore, we intend to discuss the current therapeutic approach for the management of ischemic-related pain in patients with diabetes-related CLTI through our clinical cases. The purpose of this study was to summarize different interventions available for the management of such condition, including the acceptable option for limb salvage with endovascular therapy and palliative care with pharmacotherapies in patients with CLTI.

## 2. Definition of CLTI (Formerly Known as CLI)

Despite the first definition of CLI being published in 1982, the discussion remains open about the hemodynamic criteria [13]. The emerging new definition of chronic limb-threatening ischemia (CLTI) is mainly characterized by rest pain, with or without skin ulcer or gangrene, which has replaced the term CLI in recent guidelines [5, 14]. CLTI is defined as “the presence of ischemic chronic rest pain (>2 weeks) typically in the forefoot with or without ischemic lesions or gangrene due to arterial occlusive disease” [15]. A recent position statement released by the European Society of Vascular Medicine suggests the inclusion of nonhealing leg ulceration of other origin into the definition of CLTI due to their poor prognosis and to consider the impact of frailty on adverse outcome [16].

## 3. Epidemiology of Diabetes-Related CLTI

It is estimated that up to 1 in 10 patients with LEAD has CLTI. The natural history of CTLI is of unpredictable nature and variable. Progression of CLTI from asymptomatic LEAD or IC has been estimated to be at least 5-10% within 5 years, while as much as 50% of patients diagnosed with CLTI may not even have previous history of LEAD [17]. The clinical presentation of LEAD is characteristically diffuse in distribution involving multilevel occlusions in distal vessels. In a pilot study, the prevalence of asymptomatic peripheral arterial occlusive disease in patients with diabetes was 33% [18]. LEAD has also been associated with type 2 diabetes mellitus (T2DM). Diabetes mellitus is a major global epidemic; complications of diabetes including diabetic foot ulceration are increasing proportionally. In a large cohort study of patients with diabetic foot ulceration in China, the overall amputation rate among diabetic foot patients was up to 19.03% [19]. LEAD has been found to be 2-4 times more frequent in patients with T2DM compared to the general population [8]. It was estimated that the proportional attributable fraction of T2DM for incident LEAD was 14% in the USA [20].

Majority of patients with diabetes-related CLTI may present also with nonhealing ischemic ulcer to gangrene (Fontaine stage IV) [21]. In the ADVANCE trial, including 11,140 participants who had T2DM and PAD with a median duration of seven years, the baseline prevalence of LEAD was reported at 4.6% when LEAD was defined as chronic foot ulceration due to arterial insufficiency, need for peripheral revascularization, or lower-limb amputation of at least one toe [22]. Recent research has reported higher risk of mortality from coronary arterial disease (CAD) in long-term follow-up after retrograde recanalization of chronic total occlusion (CTO) in patients with DM [23].

## 4. Pain Characteristic of Diabetes-Related CLTI

### 4.1. Different from Diabetic Neuropathy

Chronic ischemic pain is one of the most frequent causes of pain in the lower extremities [24]. In particular, the coexistence of diabetes is a significant predictor for the development of CLTI and nontraumatic amputation. Although the ischemic pain caused by CLTI has a significant neuropathic component [25, 26], there are some distinctions from those of painful diabetic neuropathy (PDN), not only in pathophysiology but also in characteristics of CLTI [27–29]. Diabetic neuropathy is a unique neurodegenerative disorder of the peripheral nervous system, of which approximately 30-50% of patients developed neuropathic pain [30]. The developing field of pain medicine has gradually revealed the pathogenesis of PDN [27]. New guidelines for the treatment of PDN using distinct classes of drugs have been issued because the pain is known to affect both the mental and physical wellbeing of patients [31]. However, the clinical characteristic of chronic ischemic pain in LEAD is diverse, ranging from asymptomatic to intermittent claudication, rest pain, nonhealing ulcers, and eventually gangrene. Both the pathophysiology and mechanism of ischemic pain remain unclear, but several mechanisms have been proposed: hemodynamic abnormalities, oxidative stress, and alterations in skeletal muscle metabolism [32]. Besides, the reduction in arterial perfusion in the affected limb leads to the accumulation of metabolites; increased acidity in the ischemic tissue and the onset of central sensitization are present in patients with CLTI [17].

The characteristic and clinical appearance of chronic ischemic pain in LEAD usually cover from nociceptive pain in patients with IC to predominantly neuropathic pain in patients with CLTI. It has been shown that questionnaires (VAS, NPSI, S-LANSS, PDI, SF-MPQ) might be a helpful tool to investigate and diagnose ischemic pain [26].

### 4.2. Different from Cancer

Previous studies have indicated that persons with diabetic lower extremity complications have 5-year mortality rates similar to many common types of cancer [33]. The impact on quality of life by poor pain management in patients with CLTI is comparable to advanced cancer patients. It is well known that managing pain is a key part of cancer treatment, and the analgesic framework ladder established by the World Health Organization (WHO) has been used to guide clinicians through a systematic approach for many years [34]. The analgesic ladder consists of a stepwise approach which includes the use of some analgesic drugs, such as nonsteroidal anti-inflammatory drugs (NSAIDs), weak opioids, and strong opioids and optional nonpharmacologic management in treating cancer pain. The effectiveness of this recommendation is confirmed in a majority of patients with cancer pain. The next question is whether a clinician can adopt this framework in the mitigation of pain for patients with diabetes-related CLTI. To the best of our knowledge, there is an ongoing debate about whether these guidelines remain the optimal pain management in all patients which encompasses persons with diabetic lower extremity complications.

## 5. Intervention of Pain Management in CLTI

### 5.1. Endovascular Therapy

In recent years, three leading vascular societies including the European Society for Vascular Surgery, the Society for Vascular Surgery, and the World Federation of Vascular Societies were determined to launch the Global Vascular Guidelines (GVS) in the effort to address the appropriate management of CLTI. Successful revascularization in CLTI, particularly in patients with tissue loss, nearly always requires reperfusion to the foot to promote wound healing and pain relief. Once the clinical manifestations of CLTI such as rest pain, ischemic ulceration, or gangrene have developed, the choice of the intervention such as balloon angioplasty, stenting, and surgical revascularization should be considered in these patients [2]. Moreover, patients who had substantial tissue loss on the background of diabetes-related CLTI will require rapid revascularization within 2 weeks from the first evaluation to in order to preserve the affected limb [35]. The following case presentations elaborate on our successful efforts in pain management and limb salvage in patients presented with tissue loss from underlying ulcerations secondary to diabetes-related CLTI.

**Figure 1:**
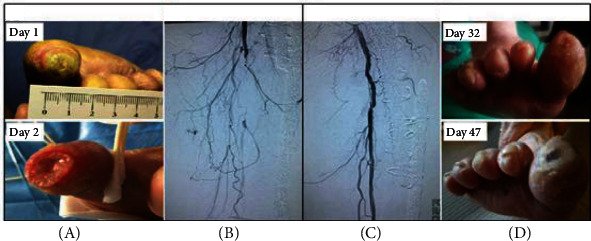
Lower limb salvage with revascularization in diabetic chronic limb-threatening ischemia.

A 68-year-old female with T2DM was admitted to the hospital with a 2-month history of progressing pain and redness in her right foot. She presented a 14-day history of worsening symptoms, especially in the big toe. Physical examination revealed a necrotic slough over the apex of the right hallux (Figure 1(a)), skin temperature was unremarkable, and pedal pulses were nonpalpable. The ankle-brachial index (ABI) was 0.4. The wound measured as 1.5 cm × 1.0 cm tissue loss without signs of bleeding (Figure 1(a)). Standard medical treatments including antibiotics were administered, blood glucose control was optimized, and peripheral circulation was improved. Analgesic medications such as ibuprofen plus codeine tablets (up to 2 tablets every 4 hours but not take more than 6 tablets in 24 hours), tramadol hydrochloride sustained release tablets, and intramuscular tramadol injection (100 mg, till a maximum of 400 mg per 24 h) were administered when necessary. However, the pain relief did not seem to be adequate, especially at night. Angiography indicated occlusion at the right anterior tibiofibular artery and segmental stenosis of the posterior tibial artery (Figure 1(b)). She underwent balloon angioplasty from the right dorsal artery to the posterior tibial artery, and intraoperative angiography showed satisfactory lumen diameter (Figure 1(c)). After 1 month, her wound recovered and the pain subsided (Figure 1(d)).

The diagnosis of CLTI was made on background of clinical symptoms of ischemic rest pain and nonhealing ulceration over two weeks, in conjunction with perfusion studies of the lower limb such as ABI and angiography. The learning point from this case is early revascularization, and appropriate analgesic medication could be an effective treatment to achieve adequate pain relief and limb salvage. This case study exemplifies the importance of revascularization in the management of pain resulted by diabetes-related CLTI.

Although revascularization strategy has been emphasized in the treatment of CLTI, the adequacy of pain management is entirely based on the drug of choice. A recent systematic review reported pharmacological therapies for the management of ischemic pain in patients with nonsalvageable CLTI [32]. Six studies were identified from 792 studies that met full inclusion criteria, and evaluated the use of intravenous lidocaine [36], oral gabapentin [37], intravenous ketamine [38, 39], and the combination of transdermal buprenorphine and epidural morphine/ropivacaine infusion [40, 41]. They found that all studies had shown an improvement in severity of ischemia pain in CLTI but with varying side effects. Therefore, no pharmacological agents can be recommended in this case because of the complex pathophysiology of pain in CLTI and limited clinical evidence [32]. Importantly, clinicians and patients should be aware of the consequences of pain syndrome in diabetes and the profound progression that can occur in the face of an ischemic limb with concomitant neuropathy masking symptoms. In another example, we present a case of progressive gangrene without a previous history of LEAD and the development of rest pain, all of which have been largely disparaged by the patient until the lower limb amputation has to be considered.

**Figure 2:**
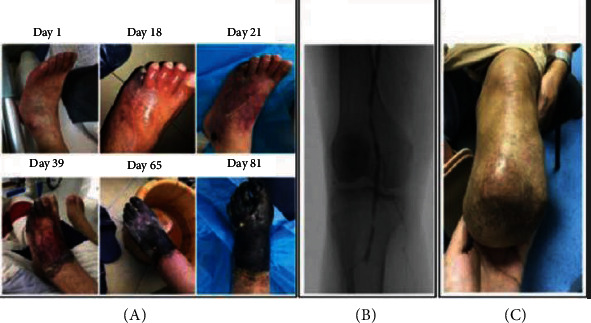
Amputation and endovascular therapy in diabetic lower limb gangrene.

A 69-year-old man with T2DM presented to our emergency department for sepsis related with the left foot. The patient had a 3-month history of a progressive ischemic lesion on his left foot, starting from mild cyanosis, nonhealing arterial ulcer to gangrene. The patient's daughter meticulously photographed the course of the lesion over 81 days (Figure 2(a), image courtesy of the patient's family). The patient has been plagued by the progressive ischemic pain over 3 months, from the tolerable rest pain to the subsequent persistent severe pain. Initial clinical presentations were signs of toes turning cyanosed with accompanying symptoms of feeling cold in his left lower limbs cold and occasional tenderness during ambulation. As the symptom was not evident, the patient paid no attention (Day 1 in Figure 2(a)). Surprisingly, after a few days, his fifth toe became gangrenous and nonhealing skin ulcer occurred on his left external ankle region (Days 18 to 21 in Figure 2(a)). At the same time, symptoms of rest pains and IC have also emerged. The pain is now characterized as a constant burning sensation or numbness in the ankle or foot in the absence of activity. He scored 4 out of 11 points on the numerical rating scale (NRS) [42]. Yet, he refused endovascular intervention or amputation of nonviable fifth toe but agreed on pain-relief medications. Unfortunately, his left foot gangrene progressed gradually upon returning home (Days 39 to 65 in Figure 2(a)). Tissue loss in the foot ranged from small ulcer to widespread gangrene. During this period, though the pain was aggravated but has been well managed by a combination of oral analgesics including acetaminophen (500 mg, pills, 2 g per 24 h) and other NSAIDs. Once again, he disparages the seriousness of the condition and had no desire to seek medical assistance. As such, we did not have an opportunity to treat until the condition was life-threating. On examination, he had profound gangrene of the left foot (Day 81 in Figure 2(a)). Following fluid resuscitation and culture of wound secretion, he was treated with broad-spectrum empiric antibiotic agents. Simultaneously, he was prescribed opioid-based analgesics such as tramadol to relieve the unbearable pain. Although it was effective by oral administration initially, the patient subsequently had an intramuscular injection of tramadol. Angiography revealed partial stenosis of the femoral artery and complete occlusion of the infrapopliteal vessels in the left lower extremity (Figure 2(b)). Following endovascular intervention and below-knee amputation, no worsening of gangrene was observed and pain has resolved completely, with no recurrence during 9 months of follow-up (Figure 2(c)).

As the risk of amputation in a deteriorating diabetic foot ulcer is high, when open or endovascular intervention has failed or is not possible, pain management is essential to improve quality of life and disease prognosis. From this case, we can learn that early medical intervention is important to improve clinical outcomes of CLTI.

Peripheral angioplasty (PTA) has been established to be the first-choice revascularization procedure in diabetic patients with CLTI. However, there are cases of CLTI that are not considered suitable candidates of angiographies or revascularizations for various reasons [43]. Firstly, it has been shown that the frailty syndrome in patients with diabetes is considered to be associated with worse prognosis for patients undergoing revascularization [44]. Secondly, on the background of chronic total occlusions (CTOs), patients with COPD treated with retrograde endovascular recanalization is associated with higher mortality [45]. Recent research has revealed that gender has an effect on long-term clinical outcomes in patients with CTOs of infrainguinal lower limb arteries treated from retrograde access with peripheral vascular interventions (PVIs) [46]. Males tend to have an increased risk of repeated PVI in patients with CTOs of infrainguinal arteries which was previously treated with retrograde access [46]. Moreover, the patients with diabetes present a higher rate of binary restenosis and amputation at 2 years following peripheral transluminal angioplasty [47] and restenosis is evident in some patients within 5 years postoperatively [48]. The rate of restenosis after endovascular treatment may be associated with impaired glycemic control and dialysis [49].

On the other hand, several studies have demonstrated that wound care, as the only treatment for CLTI, can heal approximately 50% of wounds without revascularization [50, 51]. Therefore, to some extent, it is difficult for clinicians to make the challenging decision—whether or not to perform the revascularization to save the limbs. In order to determine which patients will require and would benefit from revascularization, risk stratification that is based on three major factors as follows, Wound, Ischemia, and foot Infection (WIfI), has been introduced by the Society for Vascular Surgery Lower Extremity Threatened Limb Classification System in 2014 [12, 52]. With the WIfI classification system, revascularization significantly reduced the risk of amputation [53, 54]. This risk stratification system has been validated in clinical studies which demonstrated the potential utility of WIfI score to predict 1-year major lower extremity amputation (LEA) risk [55]. Moreover, the research also showed that after revascularization, wound severity is most strongly associated with LEA risk. Therefore, the three risk factors including tissue loss, ischemia, and infection are suggested to be evaluated to reduce the risk of amputation [56].

Endovascular therapy has increasingly become the initial clinical option for the treatment of LEAD, especially for patients with CLTI. Some recent studies have compared the clinical outcomes between open reconstruction and endovascular therapy for CLTI. The BEST-CLI (Best Endovascular versus Best Surgical Therapy in patients with Critical Limb Ischemia) trial is a prospective, multicenter, multispecialty randomized controlled trial designed to compare the effectiveness of open and endovascular interventions for 2100 patients suffering from CLTI [57–59]. In the overall CLTI population, the 3-year amputation-free survival was not different between the two treatment strategies in today's real-world settings [60].

### 5.2. Pharmacological Therapies

The treatment for CLTI is aimed at relieving ischemic pain, healing ischemic ulcers, avoiding limb loss, improving life quality, and prolonging survival. For pain management in CLTI, guidelines usually recommend a tiered approach, with a “trade off” between benefits and harms [5, 61, 62]. As no optimal pharmacological therapy has been established, the management of ischemic pain is challenging in patients unsuitable for endovascular intervention or amputation surgery [32]. It is difficult for clinicians to evaluate the effectiveness of palliative approach to deal with pain when all other options for limb salvage such as revascularization, surgery, and pharmacotherapies are exhausted. It is highlighted that intravascular lesions may be further aggravated during palliative care inadvertently. For the patients with CLTI caused by diffuse vascular calcification occlusions, endovascular therapy is ineffective and analgesia treatment cannot improve the sort of pain.

Accordingly, palliative pain management as a component of a care plan or a care focus early in the course of chronic diseases has been emphasized by the WHO [63]. Many studies have been conducted to investigate the use of lidocaine, gabapentin, or ketamine, which may optimize neuropathic pain' however, the supporting evidence of their efficacy for CLTI is limited [32, 64]. A previous study has demonstrated that patients with recurrent or stable nonhealing foot wounds can benefit from integrated palliative care such as managing pain [65]. However, it is important to stress that there is little research evaluating the risks and benefits of integrating palliative care into usual diabetic foot care, although it is possible to make some clinically meaningful recommendations. Some analgesic drugs and vasoactive substance such as tapentadol prolonged release and pentoxifylline are used to reduce the severe chronic ischemic pain with LEAD [66, 67]. Propionyl-L-carnitine (PLC) can reduce analgesic consumption and pain perception [68]. In theory, opioid combination with NSAIDs is effective at reducing opioid requirements; however, there is insufficient evidence that they can mitigate opioid side effects [34]. Nevertheless, these patients will gradually require increasing high opioid dose use [69], although some local anesthetics such as bupivacaine when combined with morphine will provide better and longer analgesic for ischemic pain as compared with a local bupivacaine alone for the short term. However, they are not used for the long term owing to serious adverse effects and potential addiction [70]. There is inconclusive evidence for the long-term effectiveness and safety of prostanoids in patients with CLTI [71, 72]. Moreover, a Cochrane review found that intravenous naftidrofuryl for CLTI was ineffective in reducing the symptoms of CLTI [73].  Table 1 shows the pharmacological therapies related to ischemic pain management in patients with CLTI.

For CLTI in patients with diabetes, in addition to the use of antithrombotic, lipid-lowering, antihypertensive, and glycemic control drugs, smoking cessation, diet, exercise, and preventive foot care advice with customized diabetic footwear are particularly important in order to achieve a better prognosis and quality of life.

### 5.3. Rehabilitative, Surgical, and Cellular Treatments

Besides the pharmacotherapies, there are many other methods that have been suggested to improve the pain and decrease medication utilization in CLTI. For example, spinal cord stimulation can provide for improvement in pain and potentiate wound healing of ischemic ulcers [74, 75]. A noncontrolled study that enrolled 38 patients with CLTI shows that 94% of patients experience pain relief [76]. The other study revealed the effectiveness of peripheral nerve crushing (Smithwick operation) to relieve chronic pain in diabetic and ischemic foot ulcers [77]. Besides chemical lumbar sympathectomy as well as epidural blockade with bupivacaine and morphine, ozone autohemotherapy seems to show beneficial effects in CLTI with ulcerations [78]. Transcutaneous electrical stimulation (TES) appears to be a useful method superior to drug therapy in curing arterial circulatory disturbances of the lower extremities [79]. Moreover, percutaneous deep vein arterialization perhaps represents an alternative option for the treatment of no-option diabetic CLI. In a pilot study including seven patients with diabetic CLI, complete wound healing was achieved in 4 of 7 patients and 5 of 7 patients at 6 and 12 months, respectively [80]. On the other hand, regenerative medicine approaches (e.g., cell and gene therapies) for CLTI have not been well established due to the restriction to rigorously conduct a randomized clinical trial. Our previous studies suggested that stem cell therapies are promising in the treatment of CLTI [81–84]. A case of DFU with normal blood supply was successfully treated with autologous platelet-rich gel combined with bone marrow mesenchymal stem cell transplantation [85]. Collectively, all these methods seem to be effective in wound healing and pain relief. However, these novel technologies should be subject to rigorous evaluation as their mechanisms and long-term outcomes remain further researched, especially in the environment of diabetic CLTI.

In summary, for patients with CLTI, endovascular therapy or surgical bypass surgery should be performed for vascular reconstruction as early as possible. Pharmacological treatments are the basis of the treatment of diabetic foot, which are suitable for patients with mild to moderate LEAD. They are primarily used to delay the development of the disease and improve the clinical symptoms and quality of life. In some cases, when the above interventions are unavailable or ineffective, some other methods such as spinal cord stimulation or lumber sympathectomy could be considered to relieve pain and to avoid complications.

**Figure 3:**
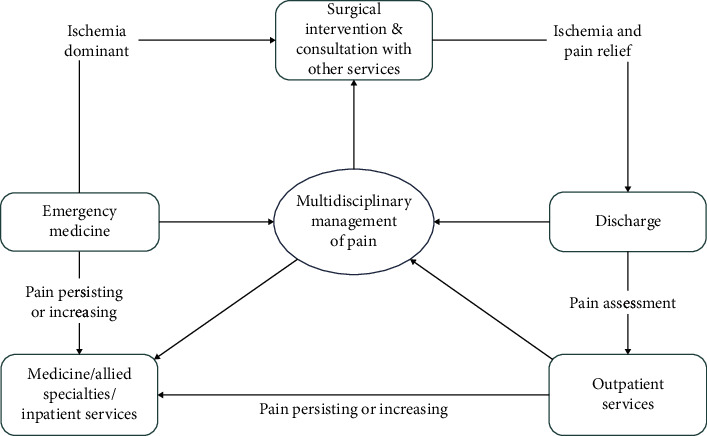
Management of ischemia pain with interdisciplinary team.

Based on our experience, a multidisciplinary team approach to manage the chronic ischemic pain is vital, since different specialties have different therapeutic options for the treatment of chronic ischemic pain [86]. Moreover, no single specialty is able to manage all aspects of the patients with diabetic CLTI. At present, there may be a potential delay from the initial clinical symptoms of pain to the subsequent referral to the appropriate medical and surgical specialties. With increased participation of multidisciplinary specialties in the pain management of diabetic CLTI, the effort to salvage the lower limb has increased significantly, which may help to improve the poor prognosis. The pain management of CLTI in patients with T2DM requires a multidisciplinary team that is composed of endocrinologists, clinical pharmacists, vascular surgeons, and podiatric surgeons.  Figure 3 illustrates a pain management team structure and the interdisciplinary components.

## 6. Conclusion

The management of pain in people with diabetes and CLTI remains a challenge. This is due to the complex pathophysiology of pain in CLTI, limited research base with pharmacological management, varying subjective feelings and severity of individuals, and varying degrees of pain relief for optional treatment approaches. For patients with ischemic pain caused by diabetes-related CLTI, the half-life of analgesia drug is short, so the effect is limited, and appropriate revascularization still remains an effective way to relieve pain and reduce the risk of amputation. Conservative therapy provides temporal pain relief but masks the progress of the ischemic foot and often leads to the disease deterioration. In addition, for ischemic diabetic foot with severe complications, all means may not be useful to avoid occurrence of adverse outcomes. Therefore, it is important for clinicians and patients to deepen their understanding of ischemic pain management and awareness of the possible adverse consequence as early as possible. Simultaneously, a multidisciplinary team approach to mitigate pain and reduce risk factors and comorbidities of CLTI is probably recommended. More efforts should be made to explore to formulate an effective intervention of relieving pain in patients with diabetic lower limb ischemia and to improve their quality of life avoiding the occurrence of adverse consequences.

## Figures and Tables

**Table 1:** Summary of pharmacological therapies related to ischemic pain management in patients with CLTI.

Reference	Study design	Participants	Intervention	Control	Administration method	Baseline pain scores	Postintervention pain scores	Statistical difference	Adverse effects
Tedeschi et al. [66]	Observational cohort study	*n* = 25	Tapentadol prolonged release	None	Oral administration for 3 months	Mean NRS: 7.9 ± 1.2	Mean NRS: visit 2: 5.7 ± 1.9; visit 3: 3.9 ± 2.1; visit 4: 2.8 ± 2.3	*p* < 0.01	None
The European Study Group [67]	Prospective, randomized, double-blind, placebo-controlled, parallel-group, multicentre trial	*n* = 314 (157 intervention, 157 control)	Pentoxifylline solution: 600 mg in 500 ml of saline	Saline: 500 ml	Intravenous infusions twice a day for a maximum of 21 days	Number (%) of patients VAS median Pentoxifylline: 40 (25-60) Control: 40 (25-60)	Number (%) of patients VAS median Pentoxifylline: -22 (-42-0) Control: -6 (-30-5)	Pentoxifylline vs. control: *p* < 0.001 95%	Gastrointestinal symptoms (pentoxifylline: 59 cases vs. control: 18 cases; *p* < 0.0001)
De Marchi et al. [68]	RCT	*n* = 48 (24 intervention, 24 control)	PLC solution: 600 mg in 250 ml of saline solution	Saline: 250 ml	Intravenous twice a day for 15 days	Mean VAS Intervention: 9.6 ± 0.4 Control: 9.5 ± 0.4	Mean VAS Intervention: 5.3 ± 1.2 Control: 8.8 ± 1.2	PLC vs. correspondent baseline: *p* < 0.01	None
Veroux et al. [71]	Open-label, nonrandomized study	*n* = 56 (group A: 25; group B: 31)	Iloprost: group A: a continuous 6-hour 0.5 to 2.0 ng/kg·min once daily; group B: 20 days at a mean dosage of 25 pg/d	None	Group A: IV infusion for at least 14 consecutive days; group B: a portable elastomeric infusion system	Group A: 16 Group B: 16	Number of complete pain relief Group A: 6 Group B: 11	Complete pain relief rate Group A: 6/16 (37.5) Group B: 11/16 (68.8)	Patients (40.0%) who experienced AEs. In group B, 2 of the 31 patients (6.5%) had hyperemia
Keskinbora and Aydinli [70]	RCT	*n* = 46 (32 bupivacaine alone, 14 bupivacaine plus morphine)	Bupivacaine plus morphine: 0.125% bupivacaine+10 mg morphine in 20 ml of saline	Bupivacaine alone: 0.125% bupivacaine in 20 ml of saline	Popliteal catheter consecutively	NRS scores (at rest) Bupivacaine alone: 9 ± 1; bupivacaine plus morphine: 9 ± 0.6; NRS scores (during activity) Bupivacaine alone: 9 ± 0.7 Bupivacaine plus morphine: 9 ± 0.7	NRS scores (at rest) Bupivacaine alone: 60 min: 1 ± 0.2; 8 h: 3 ± 1.1; 12 h: 3 ± 0.6 Bupivacaine plus morphine: 60 min: 1 ± 0.3; 8 h: 2 ± 0.7; 12 h: 2 ± 0.8 NRS scores (during activity) Bupivacaine alone: 60 min: 2 ± 0.1; 8 h: 4 ± 1.2; 12 h: 3 ± 0.6 Bupivacaine plus morphine: 60 min: 2 ± 0.2; 8 h: 2 ± 0.5; 12 h: 3 ± 0.6	NRS scores (at rest) Bupivacaine alone: 60 min: *p* < 0.0001; 8 h: *p* < 0.0001; 12 h: *p* < 0.0001 Bupivacaine plus morphine: 60 min: *p* < 0.0001(vs. baseline); 8 h: *p* < 0.0001 (vs. baseline or vs. bupivacaine alone); 12 h: *p* < 0.0001 (vs. baseline or vs. bupivacaine alone) NRS scores (during activity) Bupivacaine alone: 60 min: *p* < 0.0001; 8 h: *p* < 0.0001; 12 h: *p* < 0.0001 Bupivacaine plus morphine: 60 min: *p* < 0.0001 (vs. baseline); 8 h: *p* < 0.0001 (vs. baseline or vs. bupivacaine alone); 12 h: *p* < 0.0001(vs. baseline or vs. bupivacaine alone)	Nausea in bupivacaine plus morphine: *p* < 0.001 (30% vs. 0%)

RCT: randomized controlled trial; CLI: chronic limb ischemia; PLC: propionyl-L-carnitine; VAS: visual analogue scale; NRS: numerical rating scale.

## References

[1] Boyko E. J., Seelig A. D., Ahroni J. H. (2018). Limb- and person-level risk factors for lower-limb amputation in the prospective Seattle Diabetic Foot Study. *Diabetes Care*.

[2] Ouriel K. (2001). Peripheral arterial disease. *Lancet*.

[3] Fowkes F. G., Rudan D., Rudan I. (2013). Comparison of global estimates of prevalence and risk factors for peripheral artery disease in 2000 and 2010: a systematic review and analysis. *Lancet*.

[4] Trombert D., Caradu C., Brizzi V., Bérard X., Midy D., Ducasse E. (2015). Evidence for the use of drug eluting stents in below-the-knee lesions. *The Journal of Cardiovascular Surgery*.

[5] Conte M. S., Bradbury A. W., Kolh P. (2019). Global vascular guidelines on the management of chronic limb-threatening ischemia. *European Journal of Vascular and Endovascular Surgery*.

[6] Jude E. B., Oyibo S. O., Chalmers N., Boulton A. J. (2001). Peripheral arterial disease in diabetic and nondiabetic patients: a comparison of severity and outcome. *Diabetes Care*.

[7] Norman P. E., Davis W. A., Bruce D. G., Davis T. M. (2006). Peripheral arterial disease and risk of cardiac death in type 2 diabetes: the Fremantle Diabetes Study. *Diabetes Care*.

[8] Eid M. A., Mehta K. S., Goodney P. P. (2021). Epidemiology of peripheral artery disease. *Seminars in Vascular Surgery*.

[9] Teraa M., Conte M. S., Moll F. L., Verhaar M. C. (2016). Critical limb ischemia: current trends and future directions. *Journal of the American Heart Association*.

[10] Moulik P. K., Mtonga R., Gill G. V. (2003). Amputation and mortality in new-onset diabetic foot ulcers stratified by etiology. *Diabetes Care*.

[11] Armstrong D. G., Boulton A. J. M., Bus S. A. (2017). Diabetic foot ulcers and their recurrence. *The New England Journal of Medicine*.

[12] Mills JL Sr, Conte M. S., Armstrong D. G. (2014). The society for vascular surgery lower extremity threatened limb classification system: risk stratification based on wound, ischemia, and foot infection (WIfI). *Journal of Vascular Surgery*.

[13] Martini R. (2019). Current opinions about the definition of critical limb ischemia: a debate still open after three decades. *Clinical Hemorheology and Microcirculation*.

[14] Aboyans V., Ricco J. B., Bartelink M. E. L. (2018). 2017 ESC guidelines on the diagnosis and treatment of peripheral arterial diseases, in collaboration with the European Society for Vascular Surgery (ESVS). *European Heart Journal*.

[15] Nativel M., Potier L., Alexandre L. (2018). Lower extremity arterial disease in patients with diabetes: a contemporary narrative review. *Cardiovascular Diabetology*.

[16] Constans J., Bura-Rivière A., Visona A. (2019). Urgent need to clarify the definition of chronic critical limb ischemia – a position paper from the European Society for Vascular Medicine. *VASA*.

[17] Norgren L., Hiatt W. R., Dormandy J. A., Nehler M. R., Harris K. A., Fowkes F. G. R. (2007). Inter-society consensus for the management of peripheral arterial disease (TASC II). *Journal of Vascular Surgery*.

[18] Elhadd T. A., Robb R., Jung R. T., Stonebridge P. A., Belch J. J. F. (1999). Pilot study of prevalence of asymptomatic peripheral arterial occlusive disease in patients with diabetes attending a hospital clinic. *Practical Diabetes Int*.

[19] Jiang Y., Ran X., Jia L. (2015). Epidemiology of type 2 diabetic foot problems and predictive factors for amputation in China. *The International Journal of Lower Extremity Wounds*.

[20] Joosten M. M., Pai J. K., Bertoia M. L. (2012). Associations between conventional cardiovascular risk factors and risk of peripheral artery disease in men. *JAMA*.

[21] Takahara M., Iida O., Fujita Y., Haneda M. (2019). Clinical characteristics of Japanese diabetic patients with critical limb ischemia presenting Fontaine stage IV. *Diabetology International*.

[22] on behalf of the ADVANCE Collaborative Group, Mohammedi K., Woodward M. (2016). Presentations of major peripheral arterial disease and risk of major outcomes in patients with type 2 diabetes: results from the ADVANCE-ON study. *Cardiovascular Diabetology*.

[23] Wojtasik-Bakalarz J., Ruzsa Z., Rakowski T. (2019). Impact of coronary artery disease and diabetes mellitus on the long-term follow-up in patients after retrograde recanalization of the femoropopliteal arterial region. *Journal Diabetes Research*.

[24] Farber A., Eberhardt R. T. (2016). The current state of critical limb ischemia. *JAMA Surgery*.

[25] Gröne E., Üçeyler N., Abahji T. (2014). Reduced intraepidermal nerve fiber density in patients with chronic ischemic pain in peripheral arterial disease. *Pain*.

[26] Rüger L. J., Irnich D., Abahji T. N., Crispin A., Hoffmann U., Lang P. M. (2008). Characteristics of chronic ischemic pain in patients with peripheral arterial disease. *Pain*.

[27] Feldman E. L., Callaghan B. C., Pop-Busui R. (2019). Diabetic neuropathy. *Nature Reviews. Disease Primers*.

[28] Paisley P., Serpell M. (2017). Improving pain control in diabetic neuropathy. *Practitioner*.

[29] Pop-Busui R., Boulton A. J. M., Feldman E. L. (2017). Diabetic neuropathy: a position statement by the American Diabetes Association. *Diabetes Care*.

[30] Abbott C. A., Malik R. A., van Ross E. R., Kulkarni J., Boulton A. J. M. (2011). Prevalence and characteristics of painful diabetic neuropathy in a large community-based diabetic population in the U.K. *Diabetes Care*.

[31] Rolim L. C., Koga da Silva E. M., De Sá J. R., Dib S. A. (2017). A systematic review of treatment of painful diabetic neuropathy by pain phenotype versus treatment based on medical comorbidities. *Frontiers in Neurology*.

[32] Laoire Á. N., Murtagh F. E. M. (2018). Systematic review of pharmacological therapies for the management of ischaemic pain in patients with non-reconstructable critical limb ischaemia. *BMJ Supportive & Palliative Care*.

[33] Armstrong D. G., Wrobel J., Robbins J. M. (2007). Guest editorial: are diabetes-related wounds and amputations worse than cancer?. *International Wound Journal*.

[34] (2018). Pain management for patients with cancer. *CA: a Cancer Journal for Clinicians*.

[35] Noronen K., Saarinen E., Albäck A., Venermo M. (2017). Analysis of the elective treatment process for critical limb ischaemia with tissue loss: diabetic patients require rapid revascularisation. *European Journal of Vascular and Endovascular Surgery*.

[36] Vahidi E., Shakoor D., Meybodi M. A., Saeedi M. (2015). Comparison of intravenous lidocaine versus morphine in alleviating pain in patients with critical limb ischaemia. *Emergency Medicine Journal*.

[37] Morris-Stiff G., Lewis M. H. (2010). Gabapentin (Neurontin^®^) improves pain scores of patients with critical limb ischaemia: an observational study. *International Journal of Surgery*.

[38] Mitchell A. C., Fallon M. T. (2002). A single infusion of intravenous ketamine improves pain relief in patients with critical limb ischaemia: results of a double blind randomised controlled trial. *Pain*.

[39] Persson J., Hasselström J., Wiklund B., Heller A., Svensson J. O., Gustafsson L. L. (1998). The analgesic effect of racemic ketamine in patients with chronic ischemic pain due to lower extremity arteriosclerosis obliterans. *Acta Anaesthesiologica Scandinavica*.

[40] Aurilio C., Pace M. C., Passavanti M. B. (2009). Treatment of ischemic pain in patients suffering from peripheral vasculopathy with transdermal buprenorphine plus epidural morphine with ropivacaine vs. epidural morphine with ropivacaine. *Pain Practice*.

[41] Aurilio B., Pace M. C., Passavanti M. B. (2005). Transdermal buprenorphine combined with spinal morphine and naropine for pain relief in chronic peripheral vasculopathy. *Minerva Anestesiologica*.

[42] Price D. D., Bush F. M., Long S., Harkins S. W. (1994). A comparison of pain measurement characteristics of mechanical visual analogue and simple numerical rating scales. *Pain*.

[43] Reinecke H., Unrath M., Freisinger E. (2015). Peripheral arterial disease and critical limb ischaemia: still poor outcomes and lack of guideline adherence. *European Heart Journal*.

[44] Jakubiak G. K., Pawlas N., Cieślar G., Stanek A. (2020). Chronic lower extremity ischemia and its association with the frailty syndrome in patients with diabetes. *International Journal of Environmental Research and Public Health*.

[45] Ruzsa Z., Januszek R., Óriás V. (2020). Mortality and chronic obstructive pulmonary disease in patients treated with endovascular revascularization of the infra-inguinal lower limb arteries from retrograde access. *Ann Transl Med*.

[46] Pawlik A., Januszek R., Ruzsa Z. (2020). Gender differences and long-term clinical outcomes in patients with chronic total occlusions of infrainguinal lower limb arteries treated from retrograde access with peripheral vascular interventions. *Advances in Medical Sciences*.

[47] Lee M. S., Rha S. W., Han S. K. (2015). Comparison of diabetic and non-diabetic patients undergoing endovascular revascularization for peripheral arterial disease. *The Journal of Invasive Cardiology*.

[48] Faglia E., Dalla Paola L., Clerici G. (2005). Peripheral angioplasty as the first-choice revascularization procedure in diabetic patients with critical limb ischemia: prospective study of 993 consecutive patients hospitalized and followed between 1999 and 2003. *European Journal of Vascular and Endovascular Surgery*.

[49] Meloni M., Izzo V., Giurato L. (2018). Recurrence of critical limb ischemia after endovascular intervention in patients with diabetic foot ulcers. *Adv Wound Care (New Rochelle)*.

[50] Marston W. A., Davies S. W., Armstrong B. (2006). Natural history of limbs with arterial insufficiency and chronic ulceration treated without revascularization. *Journal of Vascular Surgery*.

[51] Elgzyri T., Larsson J., Thörne J., Eriksson K. F., Apelqvist J. (2013). Outcome of ischemic foot ulcer in diabetic patients who had no invasive vascular intervention. *European Journal of Vascular and Endovascular Surgery*.

[52] Mills J. L. (2014). Update and validation of the Society for Vascular Surgery wound, ischemia, and foot infection threatened limb classification system. *Seminars in Vascular Surgery*.

[53] Ward R., Dunn J., Clavijo L., Shavelle D., Rowe V., Woo K. (2017). Outcomes of critical limb ischemia in an urban, safety net hospital population with high WIfI amputation scores. *Annals of Vascular Surgery*.

[54] Robinson W. P., Loretz L., Hanesian C. (2017). Society for Vascular Surgery Wound, Ischemia, foot Infection (WIfI) score correlates with the intensity of multimodal limb treatment and patient- centered outcomes in patients with threatened limbs managed in a limb preservation center. *Journal of Vascular Surgery*.

[55] Mayor J., Chung J., Zhang Q. (2019). Using the Society for Vascular Surgery Wound, Ischemia, and foot Infection classification to identify patients most likely to benefit from revascularization. *Journal of Vascular Surgery*.

[56] Lew E. J., Mills J. L., Armstrong D. G. (2015). The deteriorating DFU: prioritising risk factors to avoid amputation. *Journal of Wound Care*.

[57] Farber A., Rosenfield K., Menard M. (2014). The BEST-CLI trial: a multidisciplinary effort to assess which therapy is best for patients with critical limb ischemia. *Techniques in Vascular and Interventional Radiology*.

[58] Menard M. T., Farber A., Assmann S. F. (2016). Design and rationale of the best endovascular versus best surgical therapy for patients with critical limb ischemia (BEST-CLI) trial. *Journal of the American Heart Association*.

[59] Mills J. L., Conte M. S., Murad M. H. (2019). Critical review and evidence implications of paclitaxel drug-eluting balloons and stents in peripheral artery disease. *Journal of Vascular Surgery*.

[60] Iida O., Takahara M., Soga Y. (2017). Three-year outcomes of surgical versus endovascular revascularization for critical limb ischemia: the SPINACH Study (Surgical Reconstruction versus Peripheral Intervention in Patients with Critical Limb Ischemia). *Circulation. Cardiovascular Interventions*.

[61] (2019). Introduction:Standards of medical care in diabetes-2019. *Diabetes Care*.

[62] Jones D. W., Farber A. (2019). Review of the global vascular guidelines on the management of chronic limb-threatening ischemia. *JAMA Surgery*.

[63] Hardiman M. C., World Health Organization Department of Global Capacities, Alter and Response (2012). World Health Organization perspective on implementation of international health regulations. *Emerging Infectious Diseases*.

[64] Helander E. M., Menard B. L., Harmon C. M. (2017). Multimodal analgesia, current concepts, and acute pain considerations. *Current Pain and Headache Reports*.

[65] Dunning T. (2016). Integrating palliative care with usual care of diabetic foot wounds. *Diabetes/Metabolism Research and Reviews*.

[66] Tedeschi A., De Bellis A., Francia P. (2018). Tapentadol Prolonged Release Reduces the Severe Chronic Ischaemic Pain and Improves the Quality of Life in Patients with Type 2 Diabetes. *Journal Diabetes Research*.

[67] The European Study Group (1995). Intravenous pentoxifylline for the treatment of chronic critical limb ischaemia. *European Journal of Vascular and Endovascular Surgery*.

[68] De Marchi S., Zecchetto S., Rigoni A. (2012). Propionyl-L-carnitine improves endothelial function, microcirculation and pain management in critical limb ischemia. *Cardiovascular Drugs and Therapy*.

[69] Itoga N. K., Sceats L. A., Stern J. R., Mell M. W. (2019). Association of opioid use and peripheral artery disease. *Journal of Vascular Surgery*.

[70] Keskinbora K., Aydinli I. (2009). Perineural morphine in patients with chronic ischemic lower extremity pain: efficacy and long-term results. *Journal of Anesthesia*.

[71] Vietto V., Franco J. V., Saenz V., Cytryn D., Chas J., Ciapponi A. (2018). Prostanoids for critical limb ischaemia. *Cochrane Database of Systematic Reviews*.

[72] Veroux P., Veroux M., Macarone M., Bonanno M. G., Tumminelli M. G. (2004). Efficacy of a novel method of intravenous infusion of the prostaglandin analogue iloprost for the treatment of lower-limb critical ischemia: an open- label, nonrandomized study in two cohorts. *Current Therapeutic Research, Clinical and Experimental*.

[73] Smith F. B., Bradbury A., Fowkes G. (2000). Intravenous naftidrofuryl for critical limb ischaemia. *Cochrane Database of Systematic Reviews*.

[74] Shamji M. F., Vos C. D., Sharan A. (2017). The advancing role of neuromodulation for the management of chronic treatment-refractory pain. *Neurosurgery*.

[75] Pedrini L., Magnoni F. (2007). Spinal cord stimulation for lower limb ischemic pain treatment. *Interactive Cardiovascular and Thoracic Surgery*.

[76] AUGUSTINSSON L. E., CARLSSON C. A., HOLM J., JIVEGARD L. (1985). Epidural electrical stimulation in severe limb ischemia. Pain relief, increased blood flow, and a possible limb-saving effect. *Annals of Surgery*.

[77] Nagasaki K., Obara H., Tanaka K., Koyano K., Asamia A., Kitagawa Y. (2016). Peripheral nerve crushing to relieve chronic pain in diabetic and ischaemic foot ulcers. *Journal of Wound Care*.

[78] de Montr A., van der Zee H., Bocci V. (2005). Major ozonated autohemotherapy in chronic limb ischemia with ulcerations. *Journal of Alternative and Complementary Medicine*.

[79] Debreceni L., Gyulai M., Debreceni A., Szabó K. (1995). Results of transcutaneous electrical stimulation (TES) in cure of lower extremity arterial disease. *Angiology*.

[80] Kum S., Tan Y. K., Schreve M. A. (2017). Midterm outcomes from a pilot study of percutaneous deep vein arterialization for the treatment of no-option critical limb ischemia. *J Endovasc Ter*.

[81] Jiang X. Y., Lu D. B., Chen B. (2012). Progress in stem cell therapy for the diabetic foot. *Diabetes Research and Clinical Practice*.

[82] Lu D., Chen B., Liang Z. (2010). Comparison of bone marrow mesenchymal stem cells with bone marrow-derived mononuclear cells for treatment of diabetic critical limb ischemia and foot ulcer: a double-blind, randomized, controlled trial. *Diabetes Research and Clinical Practice*.

[83] Lu D., Jiang Y., Deng W. (2019). Long-term outcomes of BMMSC compared with BMMNC for treatment of critical limb ischemia and foot ulcer in patients with diabetes. *Cell Transplantation*.

[84] Chen Y., Ma Y., Li N. (2018). Efficacy and long-term longitudinal follow-up of bone marrow mesenchymal cell transplantation therapy in a diabetic patient with recurrent lower limb bullosis diabeticorum. *Stem Cell Research & Therapy*.

[85] Wu Q., Lei X., Chen L. (2018). Autologous platelet-rich gel combined with in vitro amplification of bone marrow mesenchymal stem cell transplantation to treat the diabetic foot ulcer: a case report. *Ann Transl Med*.

[86] Rüger L. J., Irnich D., Grasmueller S., Lang P. M. (2008). Therapie chronischer ischämieschmerzen bei peripherer arterieller verschlusskrankheit. *Schmerz*.

